# Sarcopenia and Frailty in Patients with Metabolic Dysfunction-Associated Steatotic Liver Disease (MASLD): The Impact of Steroid Hormones

**DOI:** 10.3390/ijms27167431

**Published:** 2026-08-20

**Authors:** Triada Bali, Jennifer Lai, Niki G. Mourelatou, Aristi Saridaki, Sofia Tsitsou, Magdalini Adamantou, Aikaterini Kamiliou, Christos Chologitas, Margarita Sarri, Dimitra Pavlopoulou, Evangelos Liberopoulos, Anna Angelousi, Evangelos Cholongitas

**Affiliations:** 1 First Department of Internal Medicine, Medical School of National and Kapodistrian University of Athens, General Hospital of Athens “Laiko”, Agiou Thoma 17, 11526 Athens, Greece; triadabali@gmail.com (T.B.); nikimourelatou@yahoo.gr (N.G.M.); aristisaridaki@gmail.com (A.S.); sophie_tsitsou18@outlook.com (S.T.); magdalini_a@yahoo.gr (M.A.); aikkamiliou@gmail.com (A.K.); chrischologitas@gmail.com (C.C.); sarmargaret@gmail.com (M.S.); dimitrapavlp@gmail.com (D.P.); a.angelousi@gmail.com (A.A.); 2 Department of Medicine, Division of Gastroenterology and Hepatology, University of California-San Francisco, 400 Parnassus Avenue, Floor 7, San Francisco, CA 94143, USA; jennifer.lai@ucsf.edu; 3 First Department of Propaedeutic and Internal Medicine, Medical School of National and Kapodistrian University of Athens, General Hospital of Athens “Laiko”, Agiou Thoma 17, 11526 Athens, Greece; vaglimp@yahoo.com

**Keywords:** metabolic dysfunction- associated steatotic liver disease (MASLD), sarcopenia, dual-energy X-ray absorptiometry (DEXA), dehydroepiandrosterone sulfate (DHEAS), physical performance, short physical performance battery (SPPB), liver frailty index (LFI)

## Abstract

Metabolic dysfunction-associated steatotic liver disease (MASLD) is linked to sarcopenia and frailty, but data on white populations remain limited. This study evaluated sarcopenia, physical performance and steroid hormone associations with frailty in European MASLD patients. A cross-sectional study included 245 white MASLD patients. Clinical, biochemical, hormonal and elastography data were collected. Sarcopenia was assessed using dual-energy X-ray absorptiometry alongside mid-arm muscle circumference (MAMC), while physical performance was assessed using the liver frailty index (LFI) and the short physical performance battery (SPPB). Mean age was 56.2 years, 45.7% were male, and median liver stiffness was 5.2 kPa; 76.3% and 33.9% of patients had low MAMC and Appendicular Lean Mass Index, while 13.5% had abnormal SPPB and 16% were frail (i.e., LFI > 4.4). Poor physical performance was linked to lower dehydroepiandrosterone sulfate (DHEAS) levels using both LFI (OR: 0.98, 95% C.I.: 0.96–0.99, *p* = 0.01) and SPPB (OR: 1.14, 95% C.I.: 1.003–1.22, *p* = 0.026). Frailty assessed by LFI was associated with increased liver stiffness (OR: 1.24, 95% C.I.: 1.047–1.482, *p* = 0.013). We concurrently evaluated muscle mass and frailty for the first time in a white MASLD cohort. Lower DHEAS levels were associated with impaired physical performance. Further studies are needed to confirm these findings and their possible therapeutic implications.

## 1. Introduction

Metabolic dysfunction-associated steatotic liver disease (MASLD) is the most common cause of chronic liver disease, with a global prevalence of 38%, which is expected to increase further [1]. MASLD is characterized by hepatic steatosis with at least one component of metabolic syndrome including obesity, type 2 diabetes mellitus (T2DM), dyslipidemia and arterial hypertension, after other causes of steatosis have been excluded [2]. MASLD is considered a multisystemic disease, with several non-liver complications including cardiovascular events, chronic kidney disease, sleep-apnea syndrome, extrahepatic cancers and sarcopenia [3,4]. The latter is defined as loss of skeletal muscle mass, strength and function [5]. It was traditionally associated with ageing, but it has also been identified in people with chronic diseases, such as metabolic syndrome and T2DM [5]. A number of studies have shown that reduced muscle mass is also common among patients with MASLD with a prevalence of up to 63%, depending on the diagnostic method used to evaluate sarcopenia [4,6]. Assessment is usually based on dual-energy X-ray absorptiometry (DEXA) measurements or on the evaluation of muscle tissue at the L3 level in a computed-tomography (CT) scan [7].

Sarcopenia is closely linked to frailty, which is characterized by increased vulnerability to adverse outcomes in response to health stressors [8]. In patients with MASLD, frailty has been reported in 8% to 23% (95% CI: 3–38%) of individuals, depending on the frailty assessment tool used [9]. Even though sarcopenia and/or frailty have been associated with advanced liver fibrosis [10], their association with all-cause mortality appears to be independent of the severity of liver disease, including the stage of fibrosis [11]. However, to date, no study has specifically examined the presence of sarcopenia and frailty in the same cohort of patients with MASLD. This gap is especially important given the growing use of new treatments for MASLD, such as glucagon-like peptide-1 (GLP-1) receptor agonists, which are widely used for weight loss. Although these medications lead to significant weight reduction and improve metabolic health, there is increasing concern that part of the weight loss may include muscle mass, which could worsen underlying sarcopenia and frailty [12]. MASLD and sarcopenia/frailty share multiple pathophysiological mechanisms, indicating a potential bidirectional relationship between these conditions. Both are characterized by insulin resistance, chronic inflammation, gut microbiota alterations, physical inactivity, and hormonal imbalances, including disturbances in sex hormones [13]. Insulin regulates signaling pathways that protect skeletal muscles from protein degradation. Consequently, insulin resistance contributes to the development and progression of sarcopenia, while it promotes lipolysis, increasing the release of free fatty acids into the circulation and their subsequent transport to the liver [13].

In addition, MASLD can lead to mild portal hypertension due to narrowing of the hepatic sinusoids caused by lipid-laden, enlarged hepatocytes, which increase intrahepatic vascular resistance. These structural changes may be sufficient to cause mild splenic enlargement [14]. Immune activation, characterized by elevated IL-6 levels that tend to increase with MASLD severity is also associated with splenomegaly [15]. Splenomegaly, in turn, amplifies chronic low-grade inflammation and reduced circulating insulin-like growth factor-1 (IGF-1) levels, both of which may exacerbate insulin resistance and further perpetuate this pathogenic cycle [16]. As the growth hormone/IGF-1 axis is well known for its anabolic effects, promoting muscle remodeling and contributing to the maintenance of tissue integrity and healthy ageing, low IGF-1 levels may also contribute to the development of sarcopenia [17].

Some recent studies have identified a significant relationship between steroid hormones, such as testosterone and dehydroepiandrosterone sulfate (DHEAS) and the severity of MASLD [18,19]. In addition, a study conducted in individuals with cirrhosis evaluating sex hormones and frailty demonstrated that DHEAS was independently associated with poor physical function and frailty, whereas other sex hormones, including testosterone, showed no significant association [20]. However, no study has examined the effects of DHEAS and other steroid hormones on sarcopenia and/or frailty in MASLD patients.

Thus, the aim of this study was to evaluate the characteristics of MASLD patients with reduced muscle mass and physical performance using objective and well-established tools and, importantly, to examine specific hormonal aspects in relation to sarcopenia and frailty in MASLD patients concurrently for the first time.

## 2. Results

A total of 251 patients were evaluated, but six were excluded due to incomplete hormonal data and/or missing DEXA/frailty results. Thus, a total of 245 patients were included in the study, comprising 112 men (45.7%), with a mean age of 56.2 years. Seventy-eight patients (31.8%) had T2DM, 33.8% had arterial hypertension, 80.8% had hyperlipidemia and 49.4% were obese (BMI ≥ 30 kg/m^2^). The median controlled attenuation parameter (CAP) among the population was 281 dB/m and liver stiffness had a median value of 5.2 kPa. The baseline clinical and laboratory characteristics, as well as hormonal measurements and the sarcopenia/frailty profile are presented in Table 1. Regarding the assessment of sarcopenia and frailty, the median Mid-Arm Muscle Circumference (MAMC) was 19.9 cm, the median Appendicular Lean Mass Index (ALMI) was 6.9 kg/m^2^, while the median Short Physical Performance Battery (SPPB) score was 11 and the mean Liver Frailty Index (LFI) was 3.84. In total, 187 (76.3%) and 83 (33.9%) patients had low MAMC and ALMI respectively, 33 (13.5%) exhibited impaired physical function (i.e., SPPB < 10), and 40 (16%) were frail (i.e., LFI > 4.4).

### 2.1. Characteristics of Patients with or Without Reduced Muscle Mass

#### 2.1.1. ALMI

Our cohort was divided into two groups based on ALMI measurements: group 1 (n = 162) consisted of patients with normal ALMI, while group 2 (n = 83) included those with sarcopenia (low ALMI, i.e., men < 7.0 kg/m^2^, women < 5.5 kg/m^2^). Patients in group 1, compared to those in group 2, had significantly higher Body Mass Index (BMI) and waist circumference (WC)), and lower levels of high-density lipoproteins (HDL)), follicle-stimulating hormone (FSH)), and luteinizing hormone (LH)). Finally, patients with normal ALMI, compared to those with low ALMI, had more severe liver steatosis based on CAP measurements (Table 2). In multivariate analysis, BMI was the only variable independently associated with abnormal ALMI (OR: 1.21, 95% C.I.: 1.05–1.36, *p* < 0.001) with very good discriminative ability, as expressed by the area under the receiver operating characteristic (ROC) curve (AUC: 0.83, 95% C.I.: 0.76–0.89). The results remained consistent after stratification by sex.

#### 2.1.2. MAMC

Patients with reduced MAMC (i.e., ≤21.5 cm for women and ≤24.2 cm for men) (n = 187), compared to those with normal values (n = 58), had significantly lower BMI and WC, as well as less severe liver steatosis based on CAP measurements. Otherwise, the two groups had similar characteristics (Table 3). In multivariate analysis, WC was the only independent variable associated with reduced MAMC (OR: 1.25, 95% C.I.: 1.11–1.48, *p* = 0.003), but with low discriminative performance (AUC: 0.66, 95% C.I.: 0.59–0.72). The findings were similar when men and women were evaluated separately.

### 2.2. Characteristics of Patients Based on Frailty and Physical Function

#### 2.2.1. LFI

As handgrip strength is included in the calculation of the LFI, no additional analysis based on this variable was conducted. Instead, subjects were allocated into two distinct groups: patients with frailty (LFI > 4.4, n = 40) and patients without frailty (LFI ≤ 4.4, n = 205). Compared with non-frail individuals, frail patients were less often men and were older. In addition, the former group had lower creatinine levels as well as lower DHEAS and total testosterone values, while they had more severe liver stiffness (Table 4). In multivariate logistic regression analysis, DHEAS (OR: 0.98, 95% C.I.: 0.96–0.99, *p* = 0.01) and liver stiffness (OR: 1.24, 95% C.I.: 1.047–1.482, *p* = 0.013) were the only factors independently associated with frailty, although both had relatively low discriminative performance (AUC: 0.66, 95% C.I.: 0.62–0.71 and AUC: 0.72, 95% C.I.: 0.67–0.79, respectively). After adjusting for BMI and Δ4 androstenedione, DHEAS (OR: 0.98, 95% C.I.: 0.97–0.99, *p* = 0.016) and liver stiffness (OR: 1.24, 95% C.I.: 1.017–1.512, *p* = 0.032) were again independently associated with frailty. Furthermore, DHEAS continued to be independently associated with frailty (OR: 0.98, 95% C.I.: 0.97–0.99, *p* = 0.012) when total testosterone was omitted from the multivariate analysis, whereas total testosterone was not independently associated with frailty after exclusion of DHEAS. Supplementary Table S1 presents the characteristics of men and women separately according to LFI. The above multivariate results for DHEAS were validated in the male subgroup (OR: 0.93, 95% C.I.: 0.90–0.96, *p* = 0.03; after adjustment, OR: 0.94, 95% C.I.: 0.91–0.98, *p* = 0.04), while no factors were associated with LFI in women.

#### 2.2.2. SPPB

Patients with abnormal SPPB (<10, n = 33), compared to those with normal SPPB (≥10, n = 212), were older, with lower creatinine levels and lower DHEAS values. Moreover, patients in this group exhibited higher insulin resistance, as determined by the Homeostatic Model Assessment of Insulin Resistance (HOMA-IR) (Table 5). In multivariate analysis, age (OR: 0.92, 95% C.I.: 0.88–0.97, *p* = 0.026) and DHEAS (OR: 1.14, 95% C.I.: 1.003–1.22, *p* = 0.026) were the only factors independently associated with abnormal SPPB, although both had relatively low discriminative performance (AUC: 0.66, 95% C.I.: 0.62–0.71 and AUC: 0.72, 95% C.I.: 0.67–0.79, respectively). After adjusting for BMI, liver stiffness, total testosterone and Δ4 androstenedione, age (OR: 0.96, 95% C.I.: 0.93–0.99, *p* = 0.04) and DHEAS (OR: 1.12, 95% C.I.: 1.004–1.25, *p* = 0.003) were again independently associated with abnormal SPPB.

When the analysis was performed separately by sex, abnormal SPPB was only associated with decreased DHEAS levels in men, whereas in women, only older age was associated with abnormal SPPB (Supplementary Table S2).

## 3. Discussion

**Figure 1:**
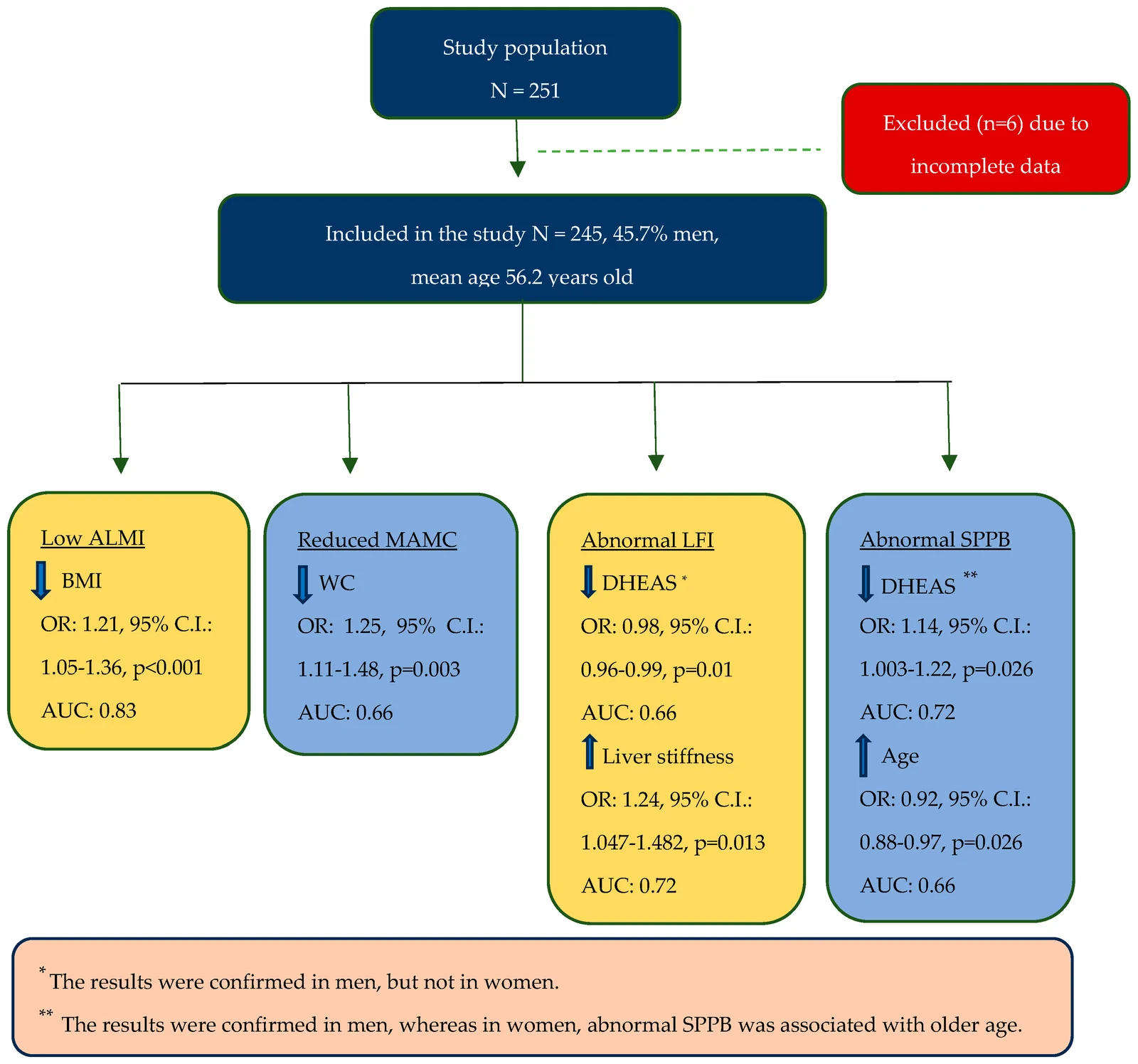
Flowchart of the study population and summary of the main findings according to muscle mass, frailty, and physical function. A total of 251 patients were evaluated, of whom 6 were excluded because of incomplete hormonal and/or DEXA or frailty data, resulting in 245 patients being included in the final analysis. Patients were categorized according to appendicular lean mass index (ALMI) mid-arm muscle circumference (MAMC), Liver Frailty Index (LFI), and Short Physical Performance Battery (SPPB) score. The figure summarizes the main associations identified in multivariate analyses. ALMI: appendicular lean mass index, AUC: area under the curve, BMI: body mass index, CAP: controlled attenuation parameter, CI: confidence interval, DHEAS: dehydroepiandrosterone sulfate, HOMA-IR: homeostatic model assessment of insulin resistance, LFI: Liver Frailty Index, MAMC: mid-arm muscle circumference, OR: odds ratio, SPPB: Short Physical Performance Battery, WC: waist circumference.

The present study extends current knowledge by, for the first time, exploring the relationship between steroid hormones and sarcopenia/frailty in white individuals with MASLD. A significant association was identified between low DHEAS levels and poor physical performance/frailty, as assessed by the SPPB and the LFI, in the total cohort and in men. Moreover, frailty, when defined by an abnormal LFI, was associated with increased liver stiffness across the entire cohort. Finally, abnormal SPPB was also associated with older age in the total cohort and in women. These findings are summarized in Figure 1. Nevertheless, despite the significant associations observed, the modest discriminative performance of these biomarkers indicates that they are currently insufficient for routine clinical prediction. Moreover, the cross-sectional design limits the above findings to associations and does not allow conclusions regarding causality.

Several studies have assessed sarcopenia in patients with MASLD within white populations using reliable and objective diagnostic methods, such as CT and/or DEXA measurements [21,22]. In addition, few studies have evaluated frailty in MASLD patients [10,23]. However, there are no studies that have jointly examined sarcopenia and frailty in the same MASLD population. In our MASLD cohort, the prevalence of sarcopenia was 33.9% when defined by ALMI and 76.3% when assessed using the MAMC. We used ALMI and MAMC to define sarcopenia, with both measures reflecting muscle quantity. This approach is not uncommon in studies of liver diseases, where muscle mass and muscle strength/physical performance are often evaluated separately [24,25].

In addition, frailty prevalence was 16% according to the LFI, while 13.5% of patients had poor physical performance based on the SPPB. Interestingly, we found that sarcopenia, as assessed by ALMI derived from DEXA and MAMC, was associated with lower BMI and reduced WC, respectively. These findings are in line with the existing literature [26,27,28]. For example, a recent large study analyzing data from adults over 20 years old from the general population reported that individuals with lower BMI were more likely to be sarcopenic, possibly because BMI is an indicator of malnutrition and chronic disease in this population [27]. In addition, in a large Chinese population-based study, it was found that sarcopenia was associated with lower BMI and reduced WC, which is also consistent with the notion that BMI/WC does not differentiate between fat and lean mass, and that a reduction in BMI/WC may reflect a loss of muscle mass [26,28].

In order to assess physical performance and frailty, we calculated the SPPB and LFI. Existing research has described a high prevalence of frailty among individuals with MASLD [10], yet the specific factors associated with frailty (e.g., age, metabolic characteristics, hormones, severity of liver fibrosis) in this population have not been systematically studied. Nevertheless, understanding these determinants is essential to identify individuals at greater risk, enabling preventive strategies and possible therapeutic interventions to mitigate frailty worsening. The need to identify the factors associated with physical performance is also important in light of emerging weight-reducing therapies that can also accelerate muscle loss, worsening sarcopenia and frailty. In our cohort, as may be expected, poor physical performance was associated with aging, since the latter is characterized by the progressive accumulation of age-related health deficits, reflecting a state of reduced physiological reserve and increased vulnerability to unfavorable outcomes in older individuals [29].

In terms of hormonal status, testosterone levels have been evaluated in patients with cirrhosis, including their association with sarcopenia [30]. Testosterone has also been evaluated in men with MASLD and its lower levels were associated with an increased risk of liver disease progression [31]. However, no previous studies have investigated the association between circulating testosterone or other androgens and sarcopenia/frailty in patients with MASLD. Interestingly, in our cohort we evaluated serum total and free testosterone, as well as DHEAS and Δ4 androstenedione and only DHEAS levels were independently associated with poor physical performance and frailty. These results align with earlier findings in older adults showing that higher DHEAS levels were associated with a lower risk of frailty [32]. Similar findings were reported in an earlier study of adults aged over 60 years, which demonstrated an inverse correlation between DHEAS levels and frailty [33], while a recent study of frailty among patients with cirrhosis confirmed these results, suggesting that this association may be relevant across diverse chronic liver disease populations [20].

To our knowledge, our study is the first to demonstrate an association between lower DHEAS levels and poor physical performance/frailty in patients with MASLD. DHEAS, an androgen precursor, is synthesized primarily in the adrenal glands, with only a small percentage being produced in the gonads. DHEAS is then desulfated and converted to Δ4 androstenedione and finally testosterone [34]. DHEAS exhibits very low affinity for sex hormone-binding globulin (SHBG), whereas testosterone binds strongly. Consequently, circulating testosterone levels are highly influenced by SHBG concentrations [35], whereas DHEAS may more accurately reflect adrenal androgen status. It should be mentioned that the independent association between DHEAS and physical performance/frailty remained significant when men were examined separately, whereas it was not observed in women. This may be explained by the fact that men generally exhibit higher DHEAS concentrations in comparison with women [36], making fluctuations in these levels potentially more impactful on men.

In our study, frailty was associated with advanced liver stiffness, and this relationship remained significant in multivariate analysis in the total cohort. There were four patients with compensated cirrhosis (i.e., liver stiffness > 12 kPa) in the study cohort. However, due to the small sample size, no subgroup analysis was performed. The majority of evidence regarding frailty is derived from cirrhotic populations, where it has been associated with progression of liver disease and worse outcomes [37]. Concerning the available literature on MASLD, similarly to our study, findings from a recent large cohort study revealed a significant relationship between the severity of hepatic fibrosis, assessed by elastography, and increased scores on the 49-item frailty index [10].

### Limitations

While the study provides valuable insights, some limitations remain. First, the number of patients included is relatively small. The majority of the participants did not have advanced fibrosis or cirrhosis related to MASLD, therefore the sample may not be fully representative of the entire spectrum of disease severity. Moreover, due to the cross-sectional nature of the study, causal relationships cannot be established. Furthermore, sarcopenia was assessed using DEXA instead of CT, which is the reference method for the evaluation of muscle mass. Nevertheless, DEXA is associated with lower radiation exposure than CT-scanning, and is generally less expensive, with prior studies demonstrating results comparable to those obtained using CT-based assessments [38]. Another limitation derives from the fact that DHEAS levels are influenced by sex and age [36]. We conducted subgroup analyses for male and female groups, while age was included in both univariate and multivariate analyses. Additionally, menstrual cycle phase was not taken into account in premenopausal women, which may have contributed to variability in hormone measurements. However, among the study population, only ten women were of premenopausal age, and when they were excluded from the analyses, the results remained the same. Nevertheless, further studies are needed to explore sex- and age- adjusted relationships. Despite the aforementioned limitations, this research offers several notable strengths. According to current evidence, this is the first study to assess sarcopenia/frailty within the same cohort of MASLD patients in a white population using objective assessment tools. Furthermore, it is the first study overall to evaluate sarcopenia/frailty in MASLD in relation to steroid androgens. Future research in larger MASLD cohorts, as well as non-MASLD populations, is needed to validate these findings and to provide additional insight.

## 4. Materials and Methods

### 4.1. Study Design and Population

All white adult patients (>18 years old) with MASLD (i.e., liver steatosis diagnosed by abdominal ultrasound using established criteria, along with at least one metabolic component) [2] were included in this cross-sectional study. Other causes of liver steatosis, such as alcohol consumption (>20 g/day for females and >30 g/day for males), viral hepatitis, autoimmune disease or drugs (e.g., corticosteroids) were ruled out. Patients with decompensated cirrhosis (i.e., ascites, variceal bleeding, encephalopathy) were also excluded. Enrollment occurred at the outpatient Hepatology clinic of the “Laiko” General Hospital of Athens, from May 2022 to May 2025.

Patients were consecutively recruited from the Hepatology clinic to minimize selection bias. Anthropometric and laboratory assessments were conducted by trained personnel to minimize measurement bias.

The study was conducted and reported according to the STROBE guidelines for observational studies. It was not prospectively registered in a public trial registry.

### 4.2. Clinical Data and Laboratory Measurements

Detailed medical histories, including demographic characteristics such as sex, age, the presence of other chronic comorbidities (T2DM, arterial hypertension, coronary artery disease, cerebrovascular, peripheral vascular disease) and drugs, were obtained from all enrolled patients. Anthropometric data including weight, height, BMI, WC, arm circumference and triceps skinfold measurement, were also recorded.

Following overnight fasting, blood samples from each patient were collected in the morning, between 08.00 and 08.30 am. Biochemical and hormonal assays were conducted in the same laboratory, under standardized morning conditions. Biochemical measurements included aspartate aminotransferase (AST), alanine aminotransferase (ALT), gamma-glutamyl transferase (G-GT), creatinine, glucose and a lipid panel including total cholesterol, low- and high-density lipoprotein cholesterol and triglycerides. Biochemical parameters were measured using automated clinical chemistry assays on the Dimension EXL with LM analyzer (Siemens Healthcare Diagnostics, Newark, NJ, USA), while complete blood count (CBC) parameters were measured using an automated hematology analyzer (XN-1000, Sysmex Corporation, Kobe, Japan), based on fluorescence flow cytometry technology.

Hormonal profile, including FSH, LH, DHEAS, SHBG, total testosterone and Δ4 androstenedione, was also evaluated. The Vermeulen formula was used to calculate free testosterone levels [39]. FSH and LH concentrations were measured via chemiluminescent assay (Liaison, DiaSorin, Saluggia, Italy). DHEAS, SHBG and testosterone were measured via electrochemiluminescence (Combass e801 Roche, Mannheim, Germany), while Δ4 androstenedione was quantified by radioimmunoassay (Demeditec Diagnostics GmbH, Kiel, Germany). HOMA-IR was also calculated [40].

### 4.3. Liver Elastography

Liver stiffness was measured using FibroScan^®^ (Echosens, Paris, France), in kilopascals (kPa) and hepatic steatosis was quantified using the CAP score (decibels per meter-dB/m), an indicator of hepatic fat content [41]. Blinded, specialized physicians carried out all assessments.

### 4.4. Evaluation of Sarcopenia

According to the latest updated definition of sarcopenia by the European Working Group on Sarcopenia in Older People (EWGSOP) in 2018, sarcopenia is characterized by low muscle strength, decreased muscle quantity/quality and impaired physical performance [5]. In our cohort, handgrip strength of the dominant hand was measured three times and the average value was recorded [5]. A calibrated pneumatic handheld dynamometer was used for this process.

Muscle quantity was evaluated by anthropometric data and DEXA. Mid upper arm circumference (MAC) (i.e., assessment conducted at the halfway point between the tip of the shoulder and the elbow) and triceps skin fold (TSF) were measured at the same site on the posterior aspect of the arm and then used for MAMC calculation through MAC (cm) − 3.14 × TSF (cm) equation. Cut-offs for moderate/severe mass depletion were ≤21.5 cm in women and ≤24.2 cm in men [42]. DEXA was used to determine the ALMI, which is the Appendicular Lean Mass (ALM) adjusted to height (ALMI = ALM/height^2^). According to EWGSOP, ALMI < 5.5 kg/m^2^ in women and ALMI < 7 kg/m^2^ in men are cutoffs to define low muscle mass [7]. Although the EWGSOP2 definition of confirmed sarcopenia requires the coexistence of low muscle strength and low muscle mass [5], in the present study we evaluated sarcopenia using quantification of muscle mass based on ALMI and MAMC.

### 4.5. Evaluation of Physical Function and Frailty

The SPPB [43] and the LFI were calculated to evaluate physical performance [44]. Gait speed, balance, and chair stand assessments were utilized to determine the SPPB score, and a threshold of <10 points was applied to define poor physical performance [43]. The LFI was derived from sex-adjusted dominant-handgrip strength, five-times chair stand duration, and a balance test. Patients with a score > 4.4 were categorized as frail [45].

### 4.6. Statistical Analysis

Statistical analyses were conducted using IBM^®^ SPSS^®^ Statistics, version 30.0. Patients with missing data were excluded. Normally distributed continuous variables were expressed as mean ± standard deviation (SD), while non-normally distributed variables were reported as medians with ranges. Comparisons between groups were performed using the Student’s *t*-test and the Mann–Whitney U-test for normally and non-normally distributed variables, respectively. Categorical variables were presented as absolute frequencies (N) and percentages (%), and group differences were analyzed using the corrected chi-square test or the two-sided Fisher’s exact test, as appropriate. A *p* value < 0.05 was considered statistically significant. Stepwise logistic regression was performed for multivariate analysis, including all variables that demonstrated statistically significant associations (*p* < 0.05) in the univariate analyses. Multivariate logistic regression models adjusted for potential confounders (such as age, BMI, serum creatinine and liver stiffness) were also applied. When multiple hormones belonging to the same endocrine axis were significant in univariate analyses, multivariate analyses were adjusted for all hormones within that axis, regardless of their univariate significance. The discriminative performance of the independent variables that were derived from multivariate analysis was assessed using the AUC [46].

## 5. Conclusions

In conclusion, sarcopenia in patients with MASLD, when assessed using anthropometric and DEXA measurements, appeared to be independently associated with lower BMI and WC. Frailty in our MASLD cohort, assessed for the first time in relation to hormonal status, was independently associated with lower circulating DHEAS concentrations in the total cohort and in men, but not women, as well as with more advanced liver stiffness. We hope that future prospective research across larger and more varied populations, will help to validate these findings, to demonstrate causality and to better define potential targets for prevention and therapeutic intervention.

## Figures and Tables

**Table 1:** Clinical and laboratory characteristics, sarcopenia and frailty in 245 patients with MASLD.

Variable	N = 245
Sex, male (n%)	112 (45.7)
Age, years	56.18 ± 13.46
BMI, kg/m^2^	31.2 (20.4–51)
Waist circumference, cm	105.1± 14.45
Diabetes mellitus, n (%)	78 (31.8%)
Arterial hypertension, n (%)	83 (33.8%)
Obesity, n (%)	121 (49.4%)
Dyslipidemia, n (%)	198 (80.8%)
ALT, IU/L	28 (7–308)
GGT, U/L	34.5 (7–556)
Creatinine, mg/dL	0.79 (0.35–1.64)
Glucose, mg/dL	88 (45–264)
HOMA-IR	3.1 (0.2–29.6)
CHOL, mg/dL	190 ± 40.6
LDL, mg/dL	111 ± 35.2
HDL, mg/dL	48.8 (29.2–105.5)
TG, mg/dL	117 (37–507)
CAP, dB/m	281 (240–400)
Liver stiffness, kPa	5.2 (2.3–49)
Hormonal profile (Ref. range)	
DHEAS, μg/dL (33.6–249)	112 (1.08–1000)
SHBG, nmol/L (18.3–54.1)	41.45 (10.30–110)
LH, mIU/mL (2.8–6.8)	9.8 (0.11–74.53)
FSH, mIU/mL (1.3–11.8)	27.1 (0.31–177.6)
Testosterone, ng/mL (2.49–8.36)	0.51 (0.03–38.8)
Free testosterone, % (0.2–0.62)	1.61 (0.78–3.23)
Δ4, androstenedione, ng/mL (0.6–3.10)	0.71 (0.1–4.11)
Sarcopenia and frailty profile	
Handgrip strength, kg	20 (2–112)
MAMC, cm	19.9 (6.9–85.6)
ALMI, kg/m^2^	6.9 (1.2–47)
SPPB	11 (7–12)
LFI	3.84 ± 0.6

Values are presented as mean ± SD or median values [range]. Abbreviations: ALMI: Appendicular Lean Mass Index, ALT: Alanine Aminotransferase, AST: Aspartate Aminotransferase, BMI: Body Mass Index, CAP: Controlled Attenuation Parameter, CHOL: Total Cholesterol, DHEAS: Dehydroepiandrosterone Sulfate, FSH: Follicle-Stimulating Hormone, GGT: Gamma-Glutamyl Transferase, HOMA-IR: Homeostatic Model Assessment of Insulin Resistance, HDL: High-Density Lipoprotein Cholesterol, HbA1c: Glycated Hemoglobin, LFI: Liver Frailty Index, LDL: Low-Density Lipoprotein Cholesterol, LH: Luteinizing Hormone, MAMC: Mid-Arm Muscle Circumference, SHBG: Sex Hormone-Binding Globulin, SPPB: Short Physical Performance Battery, TG: Triglycerides.

**Table 2:** Clinical and laboratory characteristics of 245 patients with MASLD according to Appendicular Lean Mass Index (ALMI).

Variable	Patients with Normal ALMI (n = 162, 66.1%)	Patients with Abnormal ALMI (n = 83, 33.9%)	*p*-Value
Sex male, n, %	81 (50)	31 (33.4)	0.063
Age, years	55.54 ± 13.13	58.38 ± 14.5	0.135
BMI, kg/m^2^	33.2 (23–51)	27.4 (20.4–35.8)	<0.001
Waist circumference, cm	108.7 ± 13.7	94.43 ± 10	<0.001
ALT, IU/L	29 (8–224)	23 (7–308)	0.07
GGT, U/L	35 (7–556)	33 (7–452)	0.51
Creatinine, mg/dL	0.81 ± 0.2	0.74 ± 0.19	0.016
Glucose, mg/dL	88 (45–264)	88 (45–184)	0.95
HOMA-IR	3.1 (0.5–29.6)	3.05 (0.2–13)	0.255
CHOL, mg/dL	187.5 ± 39.4	193.2 ± 42.7	0.69
LDL, mg/dL	112 ± 35.8	108.5 ± 36	0.355
HDL, mg/dL	48 (29.2–83)	54 (30.5–105.5)	0.004
TG, mg/dL	126 ± 49.2	125.8 ± 71.1	0.354
DHEAS, μg/dL	115 (2.1–569)	102 (1.08–1000)	0.381
SHBG, nmol/L	41.3 (12.8–110)	46.1 (10.3–101)	0.118
LH, mIU/mL	8.25 (0.2–55.9)	17.3 (0.11–74.53)	0.017
FSH, mIU/mL	20.9 (2.02–168.8)	51.8 (0.31–177.6)	0.030
Testosterone, ng/mL	1.66 (0.03–38.8)	0.36 (0.03–30.2)	0.439
Free testosterone, %	1.60 (0.78–2.79)	1.51 (0.81–3.23)	0.121
Δ4, androstenedione, ng/mL	0.7 (0.1–4.11)	0.78 (0.1–3.9)	0.183
CAP, dB/m	289 (240–400)	271 (240–353)	<0.001
Liver stiffness, kPa	5.4 (2.3–49)	4.9 (3.4–21.8)	0.178

Values are presented as mean ± SD or median values [range]. Comparisons were performed using Student’s *t*-test or Mann–Whitney U test. Normality was assessed using the Shapiro–Wilk test. Abbreviations: ALMI: Appendicular Lean Mass Index, ALT: Alanine Aminotransferase, AST: Aspartate Aminotransferase, BMI: Body Mass Index, CAP: Controlled Attenuation Parameter, CHOL: Total Cholesterol, DHEAS: Dehydroepiandrosterone Sulfate, FSH: Follicle-Stimulating Hormone, GGT: Gamma-Glutamyl Transferase, HOMA-IR: Homeostatic Model Assessment of Insulin Resistance, HDL: High-Density Lipoprotein Cholesterol, HbA1c: Glycated Hemoglobin, LDL: Low-Density Lipoprotein Cholesterol, LH: Luteinizing Hormone, SHBG: Sex Hormone-Binding Globulin, TG: Triglycerides.

**Table 3:** Clinical and laboratory characteristics of 245 patients with MASLD according to Mid-Arm Muscle Circumference (MAMC).

Variable	Patients with Normal MAMC (n = 58, 23.7%)	Patients with Abnormal MAMC (n = 187, 76.3%)	*p*-Value
Sex male, n, %	32 (55)	80 (43)	0.533
Age, years	52.8 ± 12	56.5 ± 13	0.085
BMI, kg/m^2^	33.9 ± 5.8	30.9 ± 5.8	0.004
Waist circumference, cm	109 ± 12	102.4 ± 12.7	0.002
ALT, IU/L	29 (13–247)	26 (7–308)	0.065
GGT, U/L	33 (10–556)	35 (7–367)	0.541
Creatinine, mg/dL	0.8 ± 0.19	0.8 ± 0.19	0.141
Glucose, mg/dL	84 (49–153)	89 (45–264)	0.06
HOMA-IR	3.4 (0.2–12.4)	3.1 (0.5–29.6)	0.543
CHOL, mg/dL	198 ± 44	187 ± 39.4	0.105
LDL, mg/dL	122 ± 41.8	107.5 ± 33.4	0.14
HDL, mg/dL	51.1 ± 13.3	53 ± 13.9	0.414
TG, mg/dL	124 ± 43	125.5 ± 60.6	0.851
DHEAS, μg/dL	125 (15.6–608)	109 (1.08–1000)	0.267
SHBG, nmol/L	39 (12.8–97.8)	41.7 (10.3–110)	0.232
LH, mIU/mL	7.2 (1.3–44.7)	11.8 (0.11–74.53)	0.378
FSH, mIU/mL	15.4 (2.5–130.5)	41.2 (0.31–177.6)	0.059
Testosterone, ng/mL	3.07 (0.03–15.1)	0.38 (0.03–38.8)	0.178
Free testosterone, %	1.7 ± 0.43	1.64 ± 0.48	0.451
Δ4, androstenedione, ng/mL	0.67 (0.1–3.7)	0.78 (0.1–4.11)	0.517
CAP, dB/m	306 ± 45.7	279.6 ± 37	<0.001
Liver stiffness, kPa	6 (2.3–45)	4.6 (3.3–49)	0.068

Values are presented as mean ± SD or median values [range]. Comparisons were performed using Student’s *t*-test or Mann–Whitney U test. Normality was assessed using the Shapiro–Wilk test. Abbreviations: ALT: Alanine Aminotransferase, AST: Aspartate Aminotransferase, BMI: Body Mass Index, CAP: Controlled Attenuation Parameter, CHOL: Total Cholesterol, DHEAS: Dehydroepiandrosterone Sulfate, FSH: Follicle-Stimulating Hormone, GGT: Gamma-Glutamyl Transferase, HOMA-IR: Homeostatic Model Assessment of Insulin Resistance, HDL: High-Density Lipoprotein Cholesterol, HbA1c: Glycated Hemoglobin, LDL: Low-Density Lipoprotein Cholesterol, LH: Luteinizing Hormone, MAMC: Mid-Arm Muscle Circumference, SHBG: Sex Hormone-Binding Globulin, TG: Triglycerides.

**Table 4:** Clinical and laboratory characteristics of 245 patients with MASLD according to Liver Frailty Index (LFI).

Variable	Patients Without Frailty (LFI ≤ 4.4), (n = 205, 84%)	Patients with Frailty (LFI > 4.4), (n = 40, 16%)	*p*-Value
Sex male, n, %	100 (48.7)	12 (30)	0.012
Age, years	54.52 ± 12.9	61.15 ± 9.17	0.027
BMI, kg/m^2^	31.4 ± 5.2	33.72 ± 9.76	0.337
Waist circumference, cm	105.8 ± 12.35	104.65 ± 15.9	0.966
ALT, IU/L	27 (8–308)	22 (7–244)	0.450
GGT, U/L	35 (7–556)	33 (15–357)	0.972
Creatinine, mg/dL	0.83 ± 0.2	0.67 ± 0.16	0.001
Glucose, mg/dL	87 (48–264)	89.5 (45–254)	0.955
HOMA-IR	3.1 (0.2–26.2)	3.5 (0.6–29.6)	0.539
CHOL, mg/dL	190 ± 40.36	183 ± 34.34	0.448
LDL, mg/dL	112 (36–267)	107.5 (59–170)	0.280
HDL, mg/dL	53 ± 13.54	52 ± 17	0.856
TG, mg/dL	121 ± 48.6	122 ± 55.5	0.971
DHEAS, μg/dL	125 (1.08–1000)	71 (23.2–209)	0.001
SHBG, nmol/L	40.9 (10.3–110)	45.9 (15.7–85.4)	0.356
LH, mIU/mL	7.84 (0.11–55.9)	17.5 (1.62–74.53)	0.066
FSH, mIU/mL	20.1 (0.31–177.6)	59.9 (3.37–158.4)	0.084
Testosterone, ng/mL	0.51 (0.03–38.8)	0.2 (0.03–30.2)	0.024
Free testosterone, %	1.67 ± 0.45	1.55 ± 0.59	0.320
Δ4, androstenedione, ng/mL	0.72 (0.1–4.11)	0.8 (0.1–2.81)	0.843
CAP, dB/m	281.44 ± 38.88	290.65 ± 48.45	0.374
Liver stiffness, kPa	4.95 (2.3–45)	7.5 (4.6–49)	0.021

Values are presented as mean ± SD or median values [range]. Comparisons were performed using Student’s *t*-test or Mann–Whitney U test. Normality was assessed using the Shapiro–Wilk test. Abbreviations: ALT: Alanine Aminotransferase, AST: Aspartate Aminotransferase, BMI: Body Mass Index, CAP: Controlled Attenuation Parameter, CHOL: Total Cholesterol, DHEAS: Dehydroepiandrosterone Sulfate, FSH: Follicle-Stimulating Hormone, GGT: Gamma-Glutamyl Transferase, HOMA-IR: Homeostatic Model Assessment of Insulin Resistance, HDL: High-Density Lipoprotein Cholesterol, HbA1c: Glycated Hemoglobin, LFI: Liver Frailty Index, LDL: Low-Density Lipoprotein Cholesterol, LH: Luteinizing Hormone, SHBG: Sex Hormone-Binding Globulin, TG: Triglycerides.

**Table 5:** Clinical and laboratory characteristics of 245 patients with MASLD according to Short Physical Performance Battery (SPPB).

Variable	Patients with Normal SPPB (≥10 Points) (n = 212, 86.5%)	Patients with Abnormal SPPB (<10 Points) (n = 33, 13.5%)	*p*-Value
Sex male, n, %	92 (44)	20 (60.6)	0.106
Age, years	54.9 ± 13	62.43 ± 10.9	0.009
BMI, kg/m^2^	30.47 (20.4–51)	33.22 (20.68–42.45)	0.201
Waist, cm	103.45 ± 13	107.14 ± 11	0.218
ALT, IU/L	27 (8–308)	22.5 (7–78)	0.215
GGT, U/L	35 (7–556)	32.2 (11–420)	0.061
Creatinine, mg/dL	0.8 (0.35–1.37)	0.68 (0.46–1.64)	0.038
Glucose, mg/dL	88 (45–264)	94 (64–254)	0.183
HOMA-IR	3.1 (0.2–26.2)	5.1 (1.4–29.6)	0.017
CHOL, mg/dL	191 ± 41.7	181 ± 31.38	0.170
LDL, mg/dL	111 (28–267)	102 (44–196)	0.125
HDL, mg/dL	53 ± 14	53 ± 13.1	0.805
TG, mg/dL	125 ± 58.6	125 ± 49.8	0.984
DHEAS, μg/dL	120.85 (1.08–1000)	56.45 (15.6–511)	0.003
SHBG, nmol/L	39.8 (10.3–110)	52.1 (17.5–94.7)	0.530
LH, mIU/mL	8.69 (0.11–74.53)	17.6 (2.61–31.9)	0.276
FSH, mIU/mL	21.15 (0.31–177.6)	53.5 (3.37–114.8)	0.122
Testosterone, ng/mL	1.18 (0.03–38.8)	0.3 (0.03–15.1)	0.141
Free testosterone, %	1.67 ± 0.46	1.61 ± 0.52	0.658
Δ4, androstenedione, ng/mL	0.75 (0.1–4.11)	0.55 (0.1–3.89)	0.517
CAP, dB/m	285.8 ± 41.55	292.35 ± 36.73	0.504
Liver stiffness, kPa	4.95 (2.3–49)	7.4 (3.6–45)	0.064

Values are presented as mean ± SD or median values [range]. Comparisons were performed using Student’s *t*-test or Mann–Whitney U test. Normality was assessed using the Shapiro–Wilk test. Abbreviations: ALT: Alanine Aminotransferase, AST: Aspartate Aminotransferase, BMI: Body Mass Index, CAP: Controlled Attenuation Parameter, CHOL: Total Cholesterol, DHEAS: Dehydroepiandrosterone Sulfate, FSH: Follicle-Stimulating Hormone, GGT: Gamma-Glutamyl Transferase, HOMA-IR: Homeostatic Model Assessment of Insulin Resistance, HDL: High-Density Lipoprotein Cholesterol, HbA1c: Glycated Hemoglobin, LDL: Low-Density Lipoprotein Cholesterol, LH: Luteinizing Hormone, SPPB: Short Physical Performance Battery, TG: Triglycerides.

## Data Availability

The data that support the findings of this study are available from the corresponding author at cholongitas@yahoo.gr upon request, to protect confidential information.

## Supplementary Materials

The following supporting information can be downloaded at: https://www.mdpi.com/article/10.3390/ijms27167431/s1.
