# Supplementary material for: Sarcopenia and Frailty in Patients with Metabolic Dysfunction-Associated Steatotic Liver Disease (MASLD): The Impact of Steroid Hormones
Source: Int J Mol Sci. 2026 Aug 20;27(16):7431. doi: 10.3390/ijms27167431 (PMC13512960; doi:10.3390/ijms27167431)
Supplement: Supplementary file 1 [file ijms-27-07431-s001.zip › ijms-4452411-supplementary.pdf]

Table S1. Clinical and laboratory characteristics of 245 patients with MASLD separately for men and women according to Liver Frailty Index (LFI)

	**Men**			**Women**		
	Patients without frailty (n=100)	Patients with frailty (n=12)	P- value	Patients without frailty (n=105)	Patients with frailty (n=28)	P- value
Age, years	52.32±14.78	63.5±8.7	0.140	56.64±10.42	60.56±9.46	0.166
BMI, kg/m^2	30.57±4.8	39.38±5.9	0.003	32 (22-47.2)	29.67 (21.9-48)	0.539
Waist circumference, cm	106.4±11.8	119.5±10.88	0.034	101.24±12.4	99.69±14.6	0.662
ALT, IU/L	42.6±30.8	32.5±25.3	0.523	25 (8-247)	22 (7-244)	0.929
GGT, U/L	36.5 (7-556)	33.5 (15-357)	0.975	32 (7-367)	31.5 (15-269)	0.510
Creatinine, mg/dl	0.95±0.15	0.89±0.12	0.449	0.72±0.17	0.62±0.12	0.033
Glucose, mg/dl	93±32.6	130.75±96.63	0.492	90.76±20.15	90.4±30	0.949
HOMA-IR	3.1 (0.2-26.2)	3.4 (2.4-29.6)	0.425	3.47±2.89	3.23±1.98	0.793
CHOL, mg/dl	189.5±35.75	170.25±11	0.07	185 (96-344)	182 (125-248)	0.848
LDL, mg/dl	118±31.1	101±17.64	0.287	103 (36-267)	107.5 (59-170)	0.946

HDL, mg/dl	46.85 (29.5-74.3)	41.2 (34.6-68)	0.385	53.5 (29.2-94.4)	49 (33.6-105.5)	0.371
TG, mg/dl	124.5±50.2	116.25±58.2	0.751	112.5 (37-239)	115.5 (51-273)	0.805
DHEAS, µg/dL	217.1±171.28	53.35±32.52	0.004	115.47±78.44	85.14±51.95	0.143
SHBG, nmol/L	38.54±15.87	50.38±25.43	0.163	42.75 (13.1-105)	45.9 (15.7-85.4)	0.877
LH, mIU/mL	6.1±6.87	4.7±2.84	0.732	23.9±13.1	23.49±11.93	0.908
FSH, mIU/mL	6.92 (2.02-130.5)	4.97 (3.37-12)	0.479	71.63±41.54	73.27±37.89	0.887
Testosterone, ng/ml	4.29 (0.14-38.8)	3.1 (1.43-30.2)	0.354	0.19 (0.03-24)	0.15 (0.03-4.94)	0.401
Free testosterone, %	1.85±0.4	1.73±0.6	0.632	1.5 (0.78-2.61)	1.35 (0.89-3.23)	0.617
Δ4, androstenedione, ng/mL	0.75 (0.1-4.11)	1.46 (0.1-2.81)	0.922	0.9±0.85	0.88±0.66	0.889
CAP, dB/m	278.5 (240-383)	345 (290-400)	0.172	277.6±37.47	283.4±42	0.600
Liver stiffness, kPa	4.7 (2.3-45)	9.1 (7.2-49)	0.04	5.15 (3.3-14)	6.4 (4.6-13)	0.180

Values are presented as mean ± SD or median values [range].

Comparisons were performed using Student’s t-test or Mann–Whitney U test.

Normality was assessed using the Shapiro–Wilk test.

Abbreviations: ALT: Alanine Aminotransferase, AST: Aspartate Aminotransferase, BMI: Body Mass Index, CAP: Controlled Attenuation Parameter, CHOL: Total Cholesterol, DHEAS: Dehydroepiandrosterone Sulfate, FSH: Follicle-Stimulating Hormone, GGT: Gamma-Glutamyl Transferase, HOMA-IR: Homeostatic Model Assessment of Insulin Resistance, HDL: High-Density Lipoprotein Cholesterol, HbA1c: Glycated Hemoglobin, LFI: Liver Frailty Index, LDL: Low-Density Lipoprotein Cholesterol, LH: Luteinizing Hormone, SHBG: Sex Hormone-Binding Globulin, TG: Triglycerides

Table S2. Clinical and laboratory characteristics of 245 patients with MASLD separately for men and women according to Short Physical Performance Battery (SPPB)

	**Men**			**Women**		
	Patients with SPPB ≥10 points (n=92)	Patients with SPPB <10 points (n=20)	P- value	Patients with SPPB ≥10 points (n=120)	Patients with SPPB <10 points (n=13)	P- value
Age, years	52.18±15	61±12.65	0.135	57.44±10.37	63±10.45	0.048
BMI, kg/m^2	30.2 (20.45-46)	31.77 (20.68-42.45)	0.570	31.8±6.47	32.7±4.8	0.633
Waist circumference, cm	107.27±12.3	108.29±16	0.839	100±12.9	106.57±8.27	0.070
ALT, IU/L	32 (11-308)	23 (13-70)	0.380	24 (8-247)	21 (7-78)	0.603
GGT, U/L	37 (7-556)	33.5 (14-420)	0.562	32 (7-367)	31 (11-100)	0.247
Creatinine, mg/dl	0.94 (0.56-1.37)	0.81 (0.76-1.06)	0.297	0.69 (0.35-1.34)	0.65 (0.46-1.64)	0.181
Glucose, mg/dl	93±31.76	115.5±78.6	0.518	91.6±22.1	102.2±25.56	0.095

HOMA-IR	3.1 (0.2-26.2)	3.5 (3.2-29.6)	0.238	3.43±3.14	4.68±2.12	0.228
CHOL, mg/dl	190±35.7	177±30.7	0.405	188 (96-344)	177.5 (135-273)	0.400
LDL, mg/dl	119±30.6	98±36	0.113	103 (36-267)	99 (55-196)	0.694
HDL, mg/dl	45.4 (29.5-74.3)	65 (34.6-68)	0.167	56.65±15	51.91±12.5	0.237
TG, mg/dl	129.5±65.5	105±48.6	0.379	121.8±51.6	132.7±49.7	0.438
DHEAS, μg/dL	172 (2.1-1000)	53.6 (23.2-511)	0.029	85.5 (1.08-349)	74.9 (15.6-265)	0.232
SHBG, nmol/L	38.3±17.15	53.4±18.8	0.06	49.98±21.3	45.55±23.5	0.465
LH, mIU/mL	5.73±6.67	6.7±3.55	0.725	24.5±13.94	20.57±7.12	0.107
FSH, mIU/mL	6.4 (0.31-130.5)	9.87 (3.37-41.1)	0.254	70.1 (3.14-177.6)	68.9 (10.5-114.8)	0.882
Testosterone, ng/ml	4.18 (0.14-38.8)	4.2 (2.65-15.1)	0.688	0.2 (0.03-24)	0.17 (0.03-4.94)	0.573
Free testosterone, %	1.85±0.41	1.55±0.46	0.117	1.5 (0.78-3.23)	1.54 (0.92-2.79)	0.460
Δ4, androstenedione, ng/mL	0.77 (0.1-4.11)	1.34 (0.15-2.81)	0.890	0.72 (0.1-3.9)	0.49 (0.1-3.89)	0.592
CAP, dB/m	293±42.9	282±53.1	0.585	279±39.34	295.8±31.21	0.124
Liver stiffness, kPa	5 (2.3-49)	6.3 (3.6-45)	0.712	4.6 (3.3-14)	7.4 (4.3-13)	0.057

Values are presented as mean ± SD or median values [range].

Comparisons were performed using Student's t-test or Mann–Whitney U test.

Normality was assessed using the Shapiro–Wilk test.

Abbreviations: ALT: Alanine Aminotransferase, AST: Aspartate Aminotransferase, BMI: Body Mass Index, CAP: Controlled Attenuation Parameter, CHOL: Total Cholesterol, DHEAS: Dehydroepiandrosterone Sulfate, FSH: Follicle-Stimulating Hormone, GGT: Gamma-Glutamyl Transferase, HOMA-IR: Homeostatic Model Assessment of Insulin Resistance, HDL: High-Density Lipoprotein Cholesterol, HbA1c: Glycated Hemoglobin, LDL: Low-Density Lipoprotein Cholesterol, LH: Luteinizing Hormone, SPPB: Short Physical Performance Battery, TG: Triglycerides
